# *Review*: Peroxisome Proliferator-Activated Receptor-γ and Its Role in the Development and Treatment of Diabetes

**DOI:** 10.1080/15438600490451668

**Published:** 2004

**Authors:** Todd Leff, Suresh T. Mathews, Heidi S. Camp

**Affiliations:** 1 Department of Pathology and the Center for Integrative Metabolic and Endocrine Research Wayne State University School of Medicine 540 E. Canfield Detroit Michigan 48201 USA; 2 Department of Metabolic Diseases Abbott Laboratories Abbott Park Illinois USA

## Abstract

Since its identification as the receptor for antidiabetic thiazolidinedione drugs, peroxisome proliferator-activated receptor-*γ* (PPAR*γ*) has been the focus of pharmaceutical drug discovery programs directed toward finding better drugs for the treatment of diabetes, as well as the object of basic research aimed at understanding its role in the regulation of metabolism. We now understand a great deal about the crucial role that PPAR*γ* plays in adipocyte differentiation and development, and are rapidly gaining knowledge about the role of the receptor in the regulation of metabolism. However, many crucial aspects of the molecular mechanism by which modulation of PPAR*γ* activity affects insulin resistance and glucose homeostasis are still not clearly understood. Here the authors review the current status of PPAR*γ* research, with an emphasis on its role in the causes and treatment of type 2 diabetes.


*Experimental Diab. Res.*, 5:99–109, 2004
Copyright © Taylor and Francis Inc.
ISSN: 1543-8600 print / 1543-8619 online
DOI: 10.1080/15438600490451668

### *Review*: Peroxisome Proliferator-Activated Receptor-$\gamma$ and Its Role in the Development and Treatment of Diabetes

**Todd Leff, Suresh T. Mathews, and Heidi S. Camp**

*Department of Pathology and the Center for Integrative Metabolic and Endocrine Research, Wayne State University School of Medicine, Detroit, Michigan, USA; and Department of Metabolic Diseases, Abbott Laboratories, Abbott Park, Illinois, USA*

**Since its identification as the receptor for antidiabetic thiazolidinedione drugs, peroxisome proliferator-activated receptor-$\gamma$ (PPAR$\gamma$) has been the focus of pharmaceutical drug discovery programs directed toward finding better drugs for the treatment of diabetes, as well as the object of basic research aimed at understanding its role in the regulation of metabolism. We now understand a great deal about the crucial role that PPAR$\gamma$ plays in adipocyte differentiation and development, and are rapidly gaining knowledge about the role of the receptor in the regulation of metabolism. However, many crucial aspects of the molecular mechanism by which modulation of PPAR$\gamma$ activity affects insulin resistance and glucose homeostasis are still not clearly understood. Here the authors review the current status of PPAR$\gamma$ research, with an emphasis on its role in the causes and treatment of type 2 diabetes.**

**Keywords** Adipocyte; Diabetes; Gene Expression; Nuclear Receptors; Thiazolidinediones

### MOLECULAR BIOLOGY OF PEROXISOME PROLIFERATOR-ACTIVATED RECEPTOR-$\gamma$ (PPAR$\gamma$)

PPAR$\gamma$, together with the closely related receptors PPAR$\alpha$ and PPAR$\delta$, comprise the PPAR subfamily of ligand-activated transcription factors. Although structurally closely related, each of the PPAR subtypes display distinct expression patterns and biological functions [1]. The endogenous ligands for all three receptors appear to be derivatives of dietary fatty acids and arachidonate metabolites [2]. PPAR$\alpha$ is expressed predominantly in liver, heart, muscle, and kidney, where it positively regulates fatty acid oxidation pathways [3]. This function of PPAR$\alpha$ is consistent with the observation that the fibrate class of lipid-lowering drugs were found to be activators of PPAR$\alpha$ [4, 5]. The function of PPAR$\delta$ is much less well understood. It is expressed in most tissues and has been implicated in lipid homeostasis, keratinocyte proliferation, and wound healing [6–8]. PPAR$\gamma$ is enriched in adipocytes (although it is present in many tissues) and can be activated by the thiazolidinedione (TZD) class of antidiabetic drugs [9]. In addition to the role of PPAR$\gamma$ in regulating metabolism and adipocyte differentiation, which is the subject of this review, molecular and genetic studies have also established roles for PPAR$\gamma$ in inflammation and atherosclerosis [10–13].

As a member of the nuclear receptor family of ligand activated transcription factors, PPAR$\gamma$ has the classical domain structure shared by most members of this family, with clearly defined activation, ligand-binding, and DNA-binding domains (Figure 1). PPAR$\gamma$ activates transcription by binding to short DNA sequence element known as a PPRE (PPAR response element) that are found in the regulatory regions of PPAR$\gamma$ target genes (i.e., genes whose transcriptional activity is regulated by PPAR$\gamma$). The active form of the receptor that binds to the PPRE is a heterodimer with another ligand-activated transcription factor called the retinoid X receptor, or RXR (Figure 1). RXR functions as a dimeric binding partner for many nuclear receptors, including the other PPARs, the retinoic acid receptor,

Received 13 December 2003; accepted 25 January 2004.
Address correspondence to Todd Leff, PhD, Department of Pathology, Wayne State University School of Medicine, 540 E. Canfield, Detroit, MI 48201, USA. E-mail: tleff@med.wayne.edu

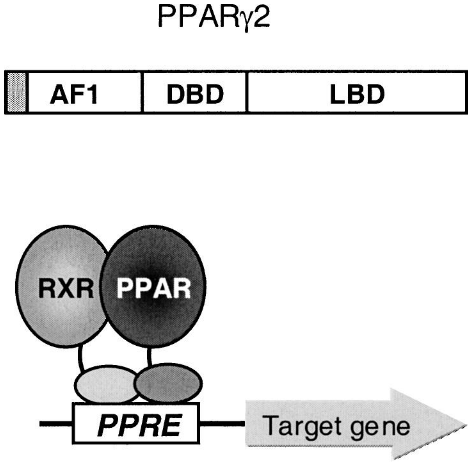

**FIGURE 1**
*Upper panel*, The domain structure of PPAR$\gamma$ showing the approximate locations of the N-terminal activation domain (AF1), the DNA binding domain (DBD), and the ligand binding domain (LBD). The shaded area indicates the 28–amino acid N-terminal domain that is present in the PPAR$\gamma$2 isoform but not in the PPAR$\gamma$1 isoform. *Lower panel*, Schematic diagram representing a PPAR$\gamma$/RXR dimer bound a PPAR-specific regulatory element (PPRE) located in the upstream regulatory region of a typical PPAR$\gamma$-regulated gene.

and the thyroid hormone receptor. Because RXR itself is also activated by specific ligands, its presence adds an additional dimension to transcriptional regulation by PPAR$\gamma$.

The molecular mechanism by which PPAR$\gamma$ activates transcription in response to thiazolidinediones is similar to how other members of the nuclear receptor family activate transcription in response to binding the appropriate ligand (for a more detailed review of the molecular mechanisms of transcriptional activation by nuclear receptors see [14]). In the absence of ligand, the transcriptionally inactive PPAR$\gamma$/RXR dimer can still bind DNA and occupy PPRE recognition sequences in PPAR target genes. In this unliganded state, the PPAR/RXR dimer can interact with one of several transcriptional accessory proteins referred to as corepressors. These corepressors proteins (e.g., N-Cor or SMRT) have histone-modifying activities that act to maintain the local chromatin configuration in a transcriptionally inactive form. The binding of a TZD (or any other agonist) to PPAR$\gamma$ induces a change in the structure of the receptor that reduces its affinity for the corepressor and increases its affinity for another class of transcriptional accessory proteins called coactivators. Transcriptional coactivators, such as P300 SRC1 or CBP, carry out various functions that increase the transcriptional activity of the PPAR target gene. Some coactivators contain a histone acetylase enzymatic activity that modifies the local chromatin structure to a more open or active form. In addition, coactivators may mediate an interaction between the receptor and the RNA polymerase complex that increases its transcriptional activity. It is the ligand-induced exchange of corepressor for coactivator proteins that leads to an increase in the transcriptional activity of PPAR$\gamma$ target genes after ligand treatment.

The PPAR$\gamma$ protein is found in tissues throughout the body, but is most highly expressed in adipose tissue. There are two major forms of the protein, PPAR$\gamma$1 and PPAR$\gamma$2, that are encoded by the same gene but transcribed from different promoters. The PPAR$\gamma$2 protein is identical to PPAR$\gamma$1 except for an additional 28 amino acids at the N-terminus in the human protein (Figure 1). The functional difference between these two PPAR$\gamma$ isoforms is not clearly understood, although as described below PPAR$\gamma$2 may have a more dominant role in regulating the process of adipocyte differentiation. It is not clear which of the two isoforms plays a more important role in mediating the therapeutic actions of the TZD drugs.

### PPAR$\gamma$ PLAYS A MAJOR ROLE IN ADIPOCYTE DIFFERENTIATION

PPAR$\gamma$ was initially recognized as a transcription factor involved in the activation of the adipocyte fatty acid binding protein aP2 during the differentiation of adipocytes from undifferentiated precursor cells [15], and it was rapidly recognized as playing a central role in the process of adipogenesis [16]. It is now known that the presence of PPAR$\gamma$ is absolutely required for the formation of adipose tissue [17]. As will be discussed later in this review, PPAR$\gamma$ (primarily the $\gamma$1 isoform) is also present at lower levels in many other tissues, and probably has important functions outside adipose tissues. On the other hand, it seems likely that many of the metabolic effects of thiazolidinedione treatment are the result of PPAR$\gamma$ activation in adipocytes. In addition, the process adipogenesis influences the number of adipocytes in the body, which together with adipocyte size, are important determinants of obesity and of multiple parameters of energy metabolism. For these reasons, the function of PPAR$\gamma$ in adipocytes is of particular significance in understanding the relationship of PPAR$\gamma$ and disease. As described below, many of the molecular details of the adipocyte differentiation process that generates mature adipocytes from fibroblast-like precursor cells are now known.

It is important to note that our understanding of how PPAR$\gamma$ participates in the generation of mature adipocytes from precursor cells is based primarily on in vitro models of adipogenesis such as the mouse 3T3L1 cell line. Although these cell lines are very amenable to experimentation, they produce adipocytes that are strikingly different in some respects than native adipocytes found in adipose tissue in vivo. For example, fully differentiated 3T3L1 adipocytes are multilocular (contain multiple lipid droplets), whereas native adipocytes in white fat

(the predominant type of adipose tissue in humans) display a unilocular distribution of lipid. Although we know that many of the characteristics of adipocyte differentiation in cultured cell lines are also important features of in vivo adipogenesis, it is important to bear in mind that some aspects of adipogenesis that have been learned from cell culture systems, as described below, may differ from the process as it occurs in vivo.

Confluent cultures of 3T3L1 fibroblasts can be induced to differentiate into adipocytes by treatment with a hormonal cocktail containing insulin, dexamethasone, and a phosphodiesterase inhibitor (for a detailed review of the molecular biology of adipogenesis see [18]). One of the first steps in this process is the reentry of growth-arrested preadipocytes into the cell cycle and the completion of several rounds of clonal expansion. Multiple genes involved in the cell-cycle control are required for this step to proceed, including the tumor suppressor retinoblastoma protein (Rb) and several cyclin-dependent kinases and their inhibitors (p18, p21, and p27). This and subsequent steps of the program of adipogenesis are controlled to a large degree by a cascade of gene expression events regulated by a small set of transcription factors. Together with PPAR$\gamma$, the three CCAAT/enhancer-binding proteins C/EBP$\alpha$, -$\beta$, and -$\delta$ comprise primary the set of transcription factor determinants of adipogenesis.

One of the initial steps in the transcriptional cascade initiated in response to adipogenic signals is the rapid induction of C/EBP$\beta$ and -$\delta$ expression. These transcription factors orchestrate cell-cycle reentry by stimulating the expression of the CDK inhibitor p21, which acts to inhibit the Rb protein and relieve its block on cell-cycle progression. C/EBP$\delta$ and -$\beta$ have also been shown to induce expression of the PPAR$\gamma$ gene that goes on to play a key role in subsequent steps in the differentiation process. The importance of C/EBP$\beta$ and -$\delta$ for adipogenesis was clearly demonstrated by loss-of-function and gain-of-function genetic studies in mice. Overexpression of either C/EBP$\beta$ or -$\delta$ in preadipocytes enhanced adipogenesis [19], whereas embryonic fibroblast cells derived from mice lacking either C/EBP$\delta$ or $\beta$ had reduced levels of adipogenesis compared with wild-type [20].

The induction of C/EBP$\beta$ and $\delta$ is immediately followed by an increase in PPAR$\gamma$ and C/EBP$\alpha$ expression. Although, as described above, the $\gamma$-1 and $\gamma$-2 isoforms are nearly identical, it appears that the two proteins have distinct activities with regard to adipocyte differentiation. When the expression of the PPAR$\gamma$2 isoform was specifically reduced by introduction of an artificial transcriptional repressor, adipogenesis was strongly inhibited [21]. A parallel experiment using a cell line in which the expression of the both isoforms was knocked down, the exogenous delivery of only the PPAR$\gamma$2 isoform into PPAR$\gamma$-deficient cells was able to completely restore the adipogenesis, whereas overexpression of only PPAR$\gamma$1 had little effect. It may be that PPAR$\gamma$1, which is already expressed in preadipocytes, behaves as a priming factor (along with C/EBP$\beta$ and -$\delta$) for the induction of PPAR$\gamma$2, and/or cell-cycle regulation to prime the preadipocytes for the differentiation.

As the program of differentiation proceeds, the expression of C/EBP$\alpha$ increases following the increase in PPAR$\gamma$2 expression. Like PPAR$\gamma$, C/EBP$\alpha$ is required for proper adipogenesis, as targeted gene knockout in mice results in embryonic lethality and failure to develop normal adipose tissue [22, 23]. There has been an intense research effort to understand the relationship between these two transcription factors and the role they play in adipogenesis. Several studies have demonstrated that PPAR$\gamma$ and C/EBP$\alpha$ coregulate each other's expression. Mice with reduced PPAR$\gamma$ expression due to heterozygous gene knockout displayed a drastically reduced level of C/EBP$\alpha$ [17], and mice with disrupted C/EBP$\alpha$ expression showed a reduced level of PPAR$\gamma$ [22]. Introduction of either PPAR$\gamma$ or C/EBP$\alpha$ into NIH3T3 cells is sufficient to convert these normally nonadipogenic cells from fibroblasts into adipocytes [16, 24]. Using cell lines missing either the PPAR$\gamma$ gene or the C/EBP$\alpha$ gene, it was shown that PPAR$\gamma$ could induce adipogenesis in the absence of C/EBP$\alpha$, but that the converse was not true [25]. Taken together, these findings support a model in which both of the transcription factors work coordinately to carry out adipogenesis; with PPAR$\gamma$2 playing the primary role and C/EBP$\alpha$ acting mostly to induce and maintain PPAR$\gamma$2 expression.

### REDUCTION OF PPAR$\gamma$ ACTIVITY BY GENETIC MUTATION CAUSES DIABETES

Genetic studies have been extremely useful in evaluating the role of PPAR$\gamma$ in obesity, lipodystrophy and type 2 diabetes. To date, a total of seven inherited mutations have been identified in the human PPAR$\gamma$ gene (Figure 2) including one

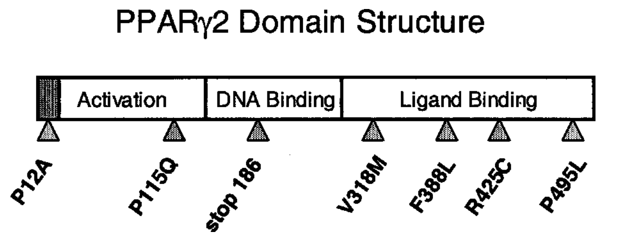

**FIGURE 2**
Location of mutation in the human PPAR$\gamma$2 gene, as described in the text. The shaded area indicates the 28–amino acid N-terminal domain that is unique to the PPAR$\gamma$2 isoform. The numbering system used to describe each mutation is based on the amino acid sequence of the PPAR$\gamma$2 isoform. Thus, V318M and P495L correspond to the V290L and P467L mutations described in Barroso et al. [27].

gain-of-function mutation (P115Q) that may be associated with obesity [26], four loss-of-function missense mutations [27–29] associated with lipodystrophy and insulin resistance, and one frameshift mutation that is associated with diabetes only as a compound heterozygote with a mutation in the protein phosphatase 1 gene [30]. These six mutations are all relatively rare, affecting very small numbers of individuals. The seventh mutation is a common polymorphism that changes a Pro to Ala at codon 12 in the N-terminus of the PPAR$\gamma$2 isoform [31], which, in some studies, has been associated with a reduced risk for type 2 diabetes and increased insulin sensitivity.

### The P12A Polymorphism in the PPAR$\gamma$2 Gene

The common P12A polymorphism in the PPAR$\gamma$ gene is a missense mutation of a CCA-to-GCA at codon 12 in the N-terminal domain that is unique to the $\gamma$-2 isoform. It appears in the human population with a frequency ranging from 12% in Caucasians to 1% in Chinese [31]. The Ala allele was associated with a reduced risk for type 2 diabetes in Finnish and Japanese populations [32], although subsequent studies failed to find an association [33, 34], or even observed an increase in body mass index in subjects with the P12A mutation [35–38]. However, a recent meta-analysis of six studies concluded that the Ala allele was generally associated with a 25% reduced risk of type 2 diabetes [39]. At the molecular level, the Ala variant showed decreased binding affinity to PPRE sequences and reduced transcriptional activity [32]. Taken together, these findings raise the possibility that a mild reduction, specifically in the activity of the PPAR$\gamma$2 isoform (with PPAR$\gamma$1 activity unchanged), causes a small increase in insulin sensitivity and confers a protection against the development of type 2 diabetes.

### PPAR$\gamma$ Mutations Associated With Familial Partial Lipodystrophy

The lipodystrophies are a heterogeneous group of adipose tissue disorders characterized by selective loss of fat from various parts of the body [40]. Dunnigan-type familial partial lipodystrophy (FPLD; MIM 151660) is an autosomal dominant syndrome characterized by total loss of subcutaneous adipose tissue selectively from extremities (visceral fat is unchanged or increased), and multiple metabolic disturbances including insulin resistance, diabetes, and dyslipidemia [40]. In some lineages mutations in *LMNA* (MIM 150330) encoding nuclear lamin A/C cause FPLD [41–43]. However, there are families with FPLD that do not have mutations in the *LMNA* gene and the genetic etiology of the syndrome in these cases is unknown. Considering the role of PPAR$\gamma$ in adipocyte biology and the development of adipose tissue, a PPAR$\gamma$ mutation would be a reasonable candidate for the genetic cause of FPLD in these cases. As outlined below, four separate mutations in the PPAR$\gamma$ gene have now been described that cause FPLD and its associated metabolic disorders.

### *P467L and V290M*

In 1999 O'Rahilly and colleagues identified two heterozygous mutations in the ligand-binding domain of PPAR$\gamma$ in three subjects with severe insulin resistance [27]. Both P467L and V290M mutations were shown to be partially defective in ligand binding and to have reduced transcriptional activity (Table 1). Further, in in vitro cell culture systems, both mutations were shown to inhibit the action of the wild-type receptor in a dominant-negative fashion. The P467L mutation involves a residue that is critical for ligand-dependent transactivation and coactivator recruitment. Consistent with its impaired transcriptional activity, the P467L mutant displays markedly attenuated release of corepressor and recruitment coactivator. Interestingly, the dominant-negative activity of both of these mutations can be alleviated by a class of tyrosine-based artificial PPAR$\gamma$ ligands, but not by TZD ligands [44]. Clinically, all three subjects show severe hyperinsulinemia, hypertension, and dyslipidemia. Given the proposed role of PPAR$\gamma$ in adipogenesis, it is important to note that all subjects have normal body mass index (BMI). Though the initial publication did not report lipoatrophy or abnormal fat distribution,

**TABLE 1**
Molecular phenotypes of PPAR$\gamma$ mutations associated with partial lipodystrophy and diabetes

| | Ligand affinity | Transcriptional activity | | | Coactivator binding | Corepressor binding |
|---|---|---|---|---|---|---|
| | | Basal | Maximal | Dom/Neg | | |
| V290M | ↓↓↓ | ↓ | Not changed | Yes | nd | nd |
| F388L | ↓ | ↓ | Not changed | No | ↓ | nd |
| R425C | ↓↓ | ↓ | ↓ | nd | nd | nd |
| P467L | ↓↓↓ | ↓ | Not changed | Yes | nd | ↑ |

*Note*. Arrows indicate approximate relative changes in activity as described in text. Dom/Neg, dominant negative; nd, not determined.

a subsequent study on the same subjects revealed a form of partial lipodystrophy in which subcutaneous fat was lost from the limbs and gluteal region but preserved in both the subcutaneous and visceral abdominal fat depots [45]. Euglycemic-hyperinsulinemic clamp studies on these individuals indicated markedly impaired insulin-induced peripheral glucose disposal and suppression of hepatic glucose output. Interestingly, the 3- and 9-year-old children of the P467L proband, who are also carriers of the P467L mutation, showed a three- to four-fold increase in fasting plasma insulin, suggesting the presence of insulin resistance. Rosiglitazone treatment of subjects with the P467L and V290M mutations for 6 months resulted in significant increases in total body fat with an improvement in plasma levels of the adipose-derived hormones leptin and Acrp30 [45]. This observation suggests that despite the dominant-negative effects of these mutations, enough PPAR$\gamma$ activity can be restored by TZD treatment to have a beneficial effect.

*R425C*

While examining PPAR$\gamma$ as a candidate gene in patients with FPLD, Agarwal and Garg [29] identified a C-to-T heterozygous mutation at nucleotide 1273 in exon 6, which changes a highly conserved residue, arginine at position 425, to cysteine (R425C) in one patient. This missense mutation was absent in DNA samples from four unaffected family members and 48 unrelated healthy individuals. No germline transmission of this mutation was demonstrated. The affected individual developed diabetes and hypertriglyceridemia at age 32 years and had a loss of subcutaneous fat from the extremities and face. Although functional aspects of this mutation have not been characterized, examining the crystal structure of PPAR$\gamma$ as determined earlier by Nolte et al. [46] suggested that Arg425 may be critical for proper protein folding.

*F388L*

Recently, a family was identified with three generations of subjects with partial lipodystrophy in whom the *LMNA* gene was normal. A mutation in the ligand-binding domain of PPAR$\gamma$ was identified that cosegregated with the lipodystrophy phenotype [28]. This novel T-to-A mutation at nucleotide 1164 in exon 5 predicted substitution of phenylalanine at codon 388 by leucine (F388L). The mutation was absent from unaffected family members and 260 normal unrelated subjects. Affected individuals had prominent muscularity of calves and lower arms due to paucity of subcutaneous fat and accumulation of subcutaneous facial, neck, suprascapular, and abdominal fat. All subjects carrying the mutation had hyperinsulinemia, hyperlipoproteinemia, and hypoalphalipoproteinemia. Functionally, this mutation impairs basal transcriptional activity and reduces affinity of the receptor for the ligand rosiglitazone. However, in contrast to the P467L and V290M mutations described above, the F388L mutation does not exert dominant-negative activity when transfected together with the wild-type protein into cultured fibroblasts.

All four of these mutations in the PPAR$\gamma$ ligand-binding domain appear to cause some degree of reduction in the activity of the receptor (Table 1). An important issue regarding the relationship between PPAR$\gamma$ function and disease is the degree to which these mutations reduce the total amount of PPAR$\gamma$ activity in cells of affected individuals. Because all of these patients are heterozygotes and carry one normal PPAR$\gamma$ gene, it is important to know if these mutations act in a dominant-negative fashion and inhibit the activity of the wild-type PPAR$\gamma$ protein present in the patient's cells. The P467L and V290M mutations have been reported to have dominant-negative activity and would be expected to reduce total PPAR$\gamma$ activity to less than 50%. On the other hand, the F388L mutation does not appear to be dominant negative, and cells from patients carrying this mutation would be expected to have 50% or more of the normal amount of PPAR$\gamma$ activity. In spite of these likely differences in total PPAR$\gamma$ activity, all of the patients have quite similar symptoms: partial lipodystrophy, insulin resistance, diabetes, dyslipidemia, and hypertension. Together, these findings suggest that even a relatively mild reduction in PPAR$\gamma$ activity is sufficient in humans to cause the striking metabolic phenotypes associated with FPLD.

However, several observations suggest that there may not be a simple relationship between a reduction in PPAR$\gamma$ activity and metabolic disease. As described above, the P12A polymorphism appears to cause a mild reduction in PPAR$\gamma$ activity, but has been associated with reduced risk of diabetes [39]. This could be due to the fact that, unlike the lipodystrophy associated mutations, the P12A change does not affect the PPAR$\gamma$1 isoform. Another possibility is that the location of the lipodystrophy mutations in ligand binding domain (as opposed to the N-terminus) has a strong effect on the physiological phenotype of the mutation. Differences in the phenotypic effects of these mutants may also be brought about by interaction with unidentified modifier genes in either the periphery or the pancreas that influence specific aspects of glucose homeostasis. Another very interesting finding that adds even more complexity is the protection from diet-induced insulin resistance seen in mice lacking one copy of the PPAR$\gamma$ gene (heterozygous PPAR$\gamma$-knockout mice) [47, 48]. The question of how a partial reduction in PPAR$\gamma$ activity leads to lipodystrophy and diabetes in humans but to protection from diabetes in mice will clearly require additional research and a better understanding of the molecular mechanism by which PPAR$\gamma$ regulates metabolism in both mice and humans.

### PPAR$\gamma$ Mutations With Unclear Phenotypes

*The P115Q Gain-of-Function Mutation*

Ristow et al. [26] reported the presence of a G-to-T mutation in exon 2 of the human PPAR$\gamma$ gene, which was present in 3% (4 of 121) of obese individuals and in 0% (0 of 237) of nonobese German Caucasians. This mutation resulted in a substitution of proline by glutamine at position 115 and prevented the phosphorylation of an adjacent serine residue at position 114 by mitogen-activated protein (MAP) kinase. Phosphorylation of PPAR$\gamma$ at this serine has been shown to negatively regulate its activity [49, 50], suggesting that the P115Q alteration would be a gain-of-function mutation. Consistent with this notion is the observation that a PPAR$\gamma$ protein bearing the P115Q mutation had increased activity in an in vitro adipocyte differentiation assay [26]. Although these results strongly suggest that the P115Q alteration acts as a gain-of-function mutation, the conclusion that the mutation causes obesity is not supported by other experiments. Results from mice bearing phosphorylation defective S114A PPAR$\gamma$ mutations were not obese and in fact were more insulin sensitive than their wild-type counterparts when subjected to a high-fat diet [51]. In addition, the P115Q mutation has failed to emerge as associated with obesity in numerous follow-up studies [52–54]. An additional recent study [55] identified the P115Q variant in one proband from 48 subjects with impaired glucose tolerance and insulin resistance (IR). This subject had a lower whole body glucose uptake characterized by euglycemic-hyperinsulinemic clamp studies and comparable body weight (BMI 28.5 kg/m$^2$) with that of the IR group (30.3 $\pm$ 0.8 kg/m$^2$). Taken together, these results do not strongly support a genetic linkage or causal connection between the P115Q mutation and obesity and diabetes in humans.

*The 186 Frameshift/Premature Stop Mutation*

The insulin resistance/diabetes syndromes described above are simple monogenic syndromes. An interesting diabetes-associated PPAR$\gamma$ mutation with a much more complex genetic profile has recently been described [30]. This frameshift mutation causes a premature stop at amino acid 186 in the DNA-binding domain that produces a completely nonfunctional receptor. Affected subjects are double heterozygotes for this mutation in the PPAR$\gamma$ gene and a mutation in the PPP1R3 gene that encodes the muscle-specific regulatory subunit of protein phosphatase 1, an enzyme that plays a key role in the regulation of glycogen storage. Individuals that had only the PPAR$\gamma$ frameshift/premature stop mutation or the PPP1RE mutation had no discernable metabolic abnormalities. However, the presence of the two mutations in the same individual resulted in severe insulin resistance, suggesting that an interaction of genes may underlie metabolic disorders such as type 2 diabetes in humans.

### ACTIVATION OF PPAR$\gamma$ BY TZD COMPOUNDS IS ANTIDIABETIC

The initial indication that PPAR$\gamma$ plays a crucial role in the regulation of glucose homeostasis and energy metabolism was the discovery that antidiabetic drugs of the TZD chemical class had their beneficial metabolic effects by direct activation of PPAR$\gamma$. In patients with type 2 diabetes, treatment with TZD compounds results in improved peripheral insulin sensitivity and reduced plasma glucose concentrations [56]. The TZDs were first identified in the 1980s, prior to the discovery of the PPAR$\gamma$ receptor and in the absence of any knowledge of their mechanism of action. In the early 1990s it was independently discovered that TZDs could cause the differentiation of 3T3L1 preadipocytes [57] and that PPAR$\gamma$ was an important transcriptional mediator of adipocyte differentiation [16, 58]. Shortly thereafter it was determined that TZDs were direct ligands of PPAR$\gamma$ [9], suggesting that the effects of these compounds on adipocytes was mediated by activation of PPAR$\gamma$ [59]. It is now generally accepted that the antidiabetic activities of the TZDs are also mediated by activation of PPAR$\gamma$. In strong support of this notion is the observation that other synthetic ligands for PPAR$\gamma$ that have been selected exclusively for their ability to activate the receptor show a very similar antidiabetic profile as the TZDs (for an example see [60]).

In spite of a great deal of work in this area, the mechanism by which TZDs increase insulin sensitivity and reduce plasma glucose concentration is not fully understood. It seems likely that the primary site of action for these drugs is adipose tissue, as adipocytes contain a higher concentration of PPAR$\gamma$ than other cell types. There are several possible mechanistic scenarios whereby TZDs could have global effects on metabolism by acting directly and uniquely in adipose tissue (discussed below). However, several lines of evidence indicate that at least some of the antidiabetic activity of TZDs is mediated by direct effects on muscle. In some studies, diabetic mice genetically engineered to have no adipose tissue still responded to TZD treatment [61], although this was not observed in all fatless mouse models [62]. In support of the possibility that TZDs have direct effects in muscle are two recent publications demonstrating that muscle-specific PPAR$\gamma$-knockout mice (animals without any expression of PPAR$\gamma$ in skeletal muscle, but with normal expression in all other tissues) are insulin resistant and at least partially defective in their response to TZD treatment [63, 64]. Although these findings do not completely agree on the relative contribution of muscle PPAR$\gamma$ to the overall action of TZD drugs, they suggest that there is a significant role for skeletal muscle PPAR$\gamma$ in the antidiabetic action of PPAR$\gamma$ agonists and that the overall physiological effect of TZD treatment is due to the combined effect of PPAR$\gamma$ activation in muscle as well as adipose tissue.

How might activation of PPAR$\gamma$ in adipose have beneficial affects on whole body insulin sensitivity? One potential mechanism is based on the known function of PPAR$\gamma$ in regulating adipocyte differentiation pathways. In this model PPAR$\gamma$ activation leads to an increase in fat-cell number and an improvement in the ability of adipocytes to store lipid. It is now understood that "ectopic" storage of fat in muscle, liver, and pancreatic islet cells is associated and may contribute to the metabolic abnormalities in these tissues associated with diabetes [65]. A TZD-induced increase in the lipid storage capability of adipose tissue would act to reduce the degree of deleterious lipid accumulation in muscle, liver, and pancreas and improve metabolic function of these tissues. In support of this model, the known target genes for PPAR$\gamma$, whose expression would presumably be activated upon TZD treatment, are involved in lipid transport and storage and would act to increase the lipid storage capability of adipose tissue. In addition, TZD treatment has been shown to induce the appearance of clusters of small multiocular adipocytes and loss of large unilocular adipocytes in diabetic mice and rats [66–68]. It is possible that these smaller adipocytes are more efficient at lipid storage then larger adipocytes. Whether the small adipocytes are derived from stem cell mitosis, recruitment of committed preadipocytes, or possibly by division of mature cells is not known. The loss of large fat cells was attributed to cellular apoptosis; however, the impact of TZD treatment on the fate and turnover of mature adipocytes has not been investigated directly [67].

Consistent with the observations of adipose tissue remodeling is the increased subcutaneous fat mass and reduced visceral fat mass seen in diabetic patients treated long-term with TZDs [69, 70]. Visceral fat is known to be more lipolytic in response to catecholamine stimulation than subcutaneous fat, and to efficiently deliver free fatty acids and other secreted factors to insulin-sensitive tissues such as liver and muscle, possibly causing an increase in insulin resistance. Although intrinsic metabolic differences between subcutaneous and visceral fat are not completely understood, current evidence suggests that subjects with increased visceral fat are at considerably higher risk for diabetes and cardiovascular complications than those with increased subcutaneous fat. These observations, plus the demonstration that PPAR$\gamma$ levels are higher in subcutaneous than in visceral fat [71], raise the possibility that PPAR$\gamma$ activation by TZDs is fat depot specific, and that differential activation of PPAR$\gamma$ in subcutaneous fat leads to a beneficial reproportioning of key metabolically active adipose beds.

Another mechanism whereby activation of PPAR$\gamma$ in adipocytes could have general effects on metabolism is by altering the production of metabolically active adipocyte-derived hormones (adipokines). In addition to its function as an energy storage depot, we now understand that adipose tissue is also a bona fide endocrine organ, secreting hormones that regulate fat metabolism in other tissues throughout the body. The list of biologically active peptides known to be secreted by fat cells has grown significantly in recent years, and although the physiological function of most of these adipokines is not fully understood, it is clear that they are important components of the system that orchestrates the control of glucose and lipid metabolism throughout the body.

Adiponectin (also called ACRP30 or adipoQ) is an excellent candidate for a fat-derived hormone that could mediate the antidiabetic effects of PPAR$\gamma$ ligands. Originally identified as a secreted fat-specific protein whose expression was induced during adipogenesis, adiponectin levels were found to be reduced in obesity and increased by weight loss. In addition, the adiponectin gene maps to a region on chromosome 3 that is associated with diabetes and metabolic syndrome (reviewed in [72]). Treatment of rodents with adiponectin was found to increase muscle fatty acid oxidation, reverse insulin resistance, and improve hepatic insulin action [73, 74]. These observations raise the possibility that the physiological role of adiponectin may be to promote lipid oxidation in nonadipose tissue, which would reduce the amount of stored lipid. Importantly, it has recently been demonstrated that levels of adiponectin are increased in patients treated with TZDs and that its expression in adipocytes is induced by PPAR$\gamma$ agonists [73–75].

Another possible adipokine that may participate in mediating the antidiabetic effects of PPAR$\gamma$ ligands is the protein resistin (also known as adipocyte secreted factor, ADSF, or FIZZ3). In contrast to adiponectin, resistin appears to have diabetes promoting effects on metabolism and was found to be overexpressed in rodent models of diet-induced obesity and to induce insulin resistance and glucose intolerance in normal mice (reviewed in [76]). These data suggest that resistin acts in a converse manner to adiponectin, increasing insulin resistance and promoting the development of diabetes. Reports that TZD treatment reduce circulating levels of resistin [77] support the possibility that it mediates some of the antidiabetic effects of these drugs. However this relationship between resistin, TZD treatment, and diabetes was not observed in all models of the disease [78, 79] and additional work will need to be carried out to fully clarify the role of resistin as another potential link between obesity and diabetes.

Yet another prodiabetic adipokine that is regulated by PPAR$\gamma$ is the inflammatory cytokine tumor necrosis factor alpha (TNF$\alpha$), which is secreted by adipocytes under some circumstances. TNF$\alpha$ production by adipocytes is elevated in obese rodents and humans and positively correlates with insulin resistance [80, 81] and in some studies inactivation of TNF$\alpha$ using antibody treatment improved insulin action [82]. As with resistin, the combination of elevated expression in obesity and insulin resistance promoting activity of TNF$\alpha$ raises the possibility that it contributes to the functional link between obesity and diabetes. Interestingly a mutual antagonism exists between TNF$\alpha$ and PPAR$\gamma$: TNF$\alpha$ inhibits PPAR$\gamma$ expression in adipocytes whereas PPAR$\gamma$ activation by TZDs can partially overcome the diabetogenic effects of TNF$\alpha$, potentially explaining at least some of the insulin-sensitizing activity of PPAR$\gamma$ ligands.

### CONCLUSION

It is clear that the PPAR$\gamma$ transcription factor carries out a variety of crucial functions in multiple tissues that influence many aspects of physiology and metabolism. However, perhaps the primary conclusions that can be drawn from a review of the current literature on the biology of PPAR$\gamma$ is that after many years of work by a large number of investigators, we still do not have anything approaching a complete understanding of the mechanisms by which this important protein carries out these functions. If there is one general lesson that can be taken from the body of literature, it is that even relatively mild alterations of PPAR$\gamma$ activity, in either direction, can have a dramatic influence on many important aspects of the physiology of the organism. This fact alone is strong motivation for continuing to work on PPAR$\gamma$ with the ultimate goals of understanding its exact role in the etiology of metabolic diseases and devising better PPAR$\gamma$-targeted therapeutic agents.

### REFERENCES

[1] Kliewer, S. A., Xu, H. E., Lambert, M. H., and Willson, T. M. (2001) Peroxisome proliferator-activated receptors: From genes to physiology. *Recent Prog. Horm. Res.*, **56,** 239–263.

[2] Michalik, L., and Wahli, W. (1999) Peroxisome proliferator-activated receptors: Three isotypes for a multitude of functions. *Curr. Opin. Biotechnol.*, **10,** 564–570.

[3] Dreyer, C., Keller, H., Mahfoudi, A., Laudet, V., Krey, G., and Wahli, W. (1993) Positive regulation of the peroxisomal beta-oxidation pathway by fatty acids through activation of peroxisome proliferator-activated receptors (PPAR). *Biol. Cell*, **77,** 67–76.

[4] Forman, B. M., Chen, J., and Evans, R. M. (1997) Hypolipidemic drugs, polyunsaturated fatty acids, and eicosanoids are ligands for peroxisome proliferator-activated receptors alpha and delta. *Proc. Natl. Acad. Sci. U.S.A.*, **94,** 4312–4317.

[5] Krey, G., Braissant, O., L'Horset, F., Kalkhoven, E., Perroud, M., Parker, M. G., and Wahli, W. (1997) Fatty acids, eicosanoids, and hypolipidemic agents identified as ligands of peroxisome proliferator-activated receptors by coactivator-dependent receptor ligand assay. *Mol. Endocrinol.*, **11,** 779–791.

[6] Peters, J. M., Lee, S. S., Li, W., Ward, J. M., Gavrilova, O., Everett, C., Reitman, M. L., Hudson, L. D., and Gonzalez, F. J. (2000) Growth, adipose, brain, and skin alterations resulting from targeted disruption of the mouse peroxisome proliferator-activated receptor beta(delta). *Mol. Cell. Biol.*, **20,** 5119–5128.

[7] Michalik, L., Desvergne, B., Tan, N. S., Basu-Modak, S., Escher, P., Rieusset, J., Peters, J. M., Kaya, G., Gonzalez, F. J., Zakany, J., Metzger, D., Chambon, P., Duboule, D., and Wahli, W. (2001) Impaired skin wound healing in peroxisome proliferator-activated receptor (PPAR)alpha and PPARbeta mutant mice. *J. Cell. Biol.*, **154,** 799–814.

[8] Oliver, W. R., Jr., Shenk, J. L., Snaith, M. R., Russell, C. S., Plunket, K. D., Bodkin, N. L., Lewis, M. C., Winegar, D. A., Sznaidman, M. L., Lambert, M. H., Xu, H. E., Sternbach, D. D., Kliewer, S. A., Hansen, B. C., and Wilson, T. M. (2001) A selective peroxisome proliferator-activated receptor delta agonist promotes reverse cholesterol transport. *Proc. Natl. Acad. Sci. U.S.A.*, **98,** 5306–5311.

[9] Lehmann, J. M., Moore, L. B., Smith-Oliver, T. A., Wilkison, W. O., Willson, T. M., and Kliewer, S. A. (1995) An antidiabetic thiazolidinedione is a high affinity ligand for peroxisome proliferator-activated receptor gamma (PPAR gamma). *J. Biol. Chem.*, **270,** 12953–12956.

[10] Ricote, M., Li, A. C., Willson, T. M., Kelly, C. J., and Glass, C. K. (1998) The peroxisome proliferator-activated receptor-gamma is a negative regulator of macrophage activation. *Nature*, **391,** 79–82.

[11] Jiang, C., Ting, A. T., and Seed, B. (1998) PPAR-gamma agonists inhibit production of monocyte inflammatory cytokines. *Nature*, **391,** 82–86.

[12] Plutzky, J. (2001) Peroxisome proliferator-activated receptors in endothelial cell biology. *Curr. Opin. Lipidol.*, **12,** 511–518.

[13] Yang, X. Y., Wang, L. H., Chen, T., Hodge, D. R., Resau, J. H., DaSilva, L., and Farrar, W. L. (2000) Activation of human T lymphocytes is inhibited by peroxisome proliferator-activated receptor gamma (PPARgamma) agonists. PPARgamma co-association with transcription factor NFAT. *J. Biol. Chem.*, **275,** 4541–4544.

[14] Xu, L., Glass, C. K., and Rosenfeld, M. G. (1999) Coactivator and corepressor complexes in nuclear receptor function. *Curr. Opin. Genet. Dev.*, **9,** 140–147.

[15] Tontonoz, P., Hu, E., Graves, R. A., Budavari, A. I., and Spiegelman, B. M. (1994) mPPAR gamma 2: Tissue-specific regulator of an adipocyte enhancer. *Genes Dev.*, **8,** 1224–1234.

[16] Tontonoz, P., Hu, E., and Spiegelman, B. M. (1994) Stimulation of adipogenesis in fibroblasts by PPAR gamma 2, a lipid-activated transcription factor. *Cell*, **79,** 1147–1156.

[17] Barak, Y., Nelson, M. C., Ong, E. S., Jones, Y. Z., Ruiz-Lozano, P., Chien, K. R., Koder, A., and Evans, R. M. (1999) PPAR gamma is required for placental, cardiac, and adipose tissue development. *Mol. Cell.*, **4,** 585–595.

[18] Rosen, E. D., and Spiegelman, B. M. (2000) Molecular regulation of adipogenesis. *Annu. Rev. Cell. Dev. Biol.*, **16,** 145–171.

[19] Darlington, G. J., Ross, S. E., and MacDougald, O. A. (1998) The role of C/EBP genes in adipocyte differentiation. *J. Biol. Chem.*, **273,** 30057–30060.

[20] Tanaka, T., Yoshida, N., Kishimoto, T., and Akira, S. (1997) Defective adipocyte differentiation in mice lacking the C/EBPbeta and/or C/EBPdelta gene. *EMBO J.*, **16,** 7432–7443.

[21] Ren, D., Collingwood, T. N., Rebar, E. J., Wolffe, A. P., and Camp, H. S. (2002) PPARgamma knockdown by engineered transcription factors: Exogenous PPARgamma2 but not PPARgamma1 reactivates adipogenesis. *Genes Dev.*, **16,** 27–32.

[22] Wu, Z., Rosen, E. D., Brun, R., Hauser, S., Adelmant, G., Troy, A. E., McKeon, C., Darlington, G. J., and Spiegelman, B. M.

(1999) Cross-regulation of C/EBP alpha and PPAR gamma controls the transcriptional pathway of adipogenesis and insulin sensitivity. *Mol. Cell*, **3,** 151–158.

[23] Wang, N. D., Finegold, M. J., Bradley, A., Ou, C. N., Abdelsayed, S. V., Wilde, M. D., Taylor, L. R., Wilson, D. R., and Darlington, G. J. (1995) Impaired energy homeostasis in C/EBP alpha knockout mice. *Science*, **269,** 1108–1112.

[24] Freytag, S. O., Paielli, D. L., and Gilbert, J. D. (1994) Ectopic expression of the CCAAT/enhancer-binding protein alpha promotes the adipogenic program in a variety of mouse fibroblastic cells. *Genes. Dev*., **8,** 1654–1663.

[25] Rosen, E. D., Hsu, C. H., Wang, X., Sakai, S., Freeman, M. W., Gonzalez, F. J., and Spiegelman, B. M. (2002) C/EBPalpha induces adipogenesis through PPARgamma: A unified pathway. *Genes Dev*., **16,** 22–26.

[26] Ristow, M., Muller-Wieland, D., Pfeiffer, A., Krone, W., and Kahn, C. R. (1998) Obesity associated with a mutation in a genetic regulator of adipocyte differentiation. *N. Engl. J. Med*., **339,** 953–959.

[27] Barroso, I., Gurnell, M., Crowley, V. E., Agostini, M., Schwabe, J. W., Soos, M. A., Maslen, G. L., Williams, T. D., Lewis, H., Schafer, A. J., Chatterjee, V. K., and O'Rahilly, S. (1999) Dominant negative mutations in human PPARgamma associated with severe insulin resistance, diabetes mellitus and hypertension. *Nature*, **402,** 880–883.

[28] Hegele, R. A., Cao, H., Frankowski, C., Mathews, S. T., and Leff, T. (2002) PPARG F388L, a transactivation-deficient mutant, in familial partial lipodystrophy. *Diabetes*, **51,** 3586–3590.

[29] Agarwal, A. K. and Garg, A. (2002) A novel heterozygous mutation in peroxisome proliferator-activated receptor-gamma gene in a patient with familial partial lipodystrophy. *J. Clin. Endocrinol. Metab*., **87,** 408–411.

[30] Savage, D. B., Agostini, M., Barroso, I., Gurnell, M., Luan, J., Meirhaeghe, A., Harding, A. H., Ihrke, G., Rajanayagam, O., Soos, M. A., George, S., Berger, D., Thomas, E. L., Bell, J. D., Meeran, K., Ross, R. J., Vidal-Puig, A., Wareham, N. J., O'Rahilly, S., Chatterjee, V. K., and Schafer, A. J. (2002) Digenic inheritance of severe insulin resistance in a human pedigree. *Nat. Genet*., **31,** 379–384.

[31] Yen, C. J., Beamer, B. A., Negri, C., Silver, K., Brown, K. A., Yarnall, D. P., Burns, D. K., Roth, J., and Shuldiner, A. R. (1997) Molecular scanning of the human peroxisome proliferator activated receptor gamma (hPPAR gamma) gene in diabetic Caucasians: Identification of a Pro12Ala PPAR gamma 2 missense mutation. *Biochem. Biophys. Res. Commun*., **241,** 270–274.

[32] Deeb, S. S., Fajas, L., Nemoto, M., Pihlajamaki, J., Mykkanen, L., Kuusisto, J., Laakso, M., Fujimoto, W., and Auwerx, J. (1998) A Pro12Ala substitution in PPARgamma2 associated with decreased receptor activity, lower body mass index and improved insulin sensitivity. *Nat. Genet*., **20,** 284–287.

[33] Hamann, A., Munzberg, H., Buttron, P., Busing, B., Hinney, A., Mayer, H., Siegfried, W., Hebebrand, J., and Greten, H. (1999) Missense variants in the human peroxisome proliferator-activated receptor-gamma2 gene in lean and obese subjects. *Eur. J. Endocrinol*., **141,** 90–92.

[34] Clement, K., Hercberg, S., Passinge, B., Galan, P., Varroud-Vial, M., Shuldiner, A. R., Beamer, B. A., Charpentier, G., Guy-Grand, B., Froguel, P., and Vaisse, C. (2000) The Pro115Gln and Pro12Ala PPAR gamma gene mutations in obesity and type 2 diabetes. *Int. J. Obes. Relat. Metab. Disord*., **24,** 391–393.

[35] Beamer, B. A., Yen, C. J., Andersen, R. E., Muller, D., Elahi, D., Cheskin, L. J., Andres, R., Roth, J., and Shuldiner, A. R. (1998) Association of the Pro12Ala variant in the peroxisome proliferator-activated receptor-gamma2 gene with obesity in two Caucasian populations. *Diabetes*, **47,** 1806–1808.

[36] Cole, S. A., Mitchell, B. D., Hsueh, W. C., Pineda, P., Beamer, B. A., Shuldiner, A. R., Comuzzie, A. G., Blangero, J., and Hixson, J. E. (2000) The Pro12Ala variant of peroxisome proliferator-activated receptor-gamma2 (PPAR-gamma2) is associated with measures of obesity in Mexican Americans. *Int. J. Obes. Relat. Metab. Disord*., **24,** 522–524.

[37] Valve, R., Sivenius, K., Miettinen, R., Pihlajamaki, J., Rissanen, A., Deeb, S. S., Auwerx, J., Uusitupa, M., and Laakso, M. (1999) Two polymorphisms in the peroxisome proliferator-activated receptor-gamma gene are associated with severe overweight among obese women. *J. Clin. Endocrinol. Metab*., **84,** 3708–3712.

[38] Stumvoll, M., and Haring, H. (2002) The peroxisome proliferator-activated receptor-gamma2 Pro12Ala polymorphism. *Diabetes*, **51,** 2341–2347.

[39] Altshuler, D., Hirschhorn, J. N., Klannemark, M., Lindgren, C. M., Vohl, M. C., Nemesh, J., Lane, C. R., Schaffner, S. F., Bolk, S., Brewer, C., Tuomi, T., Gaudet, D., Hudson, T. J., Daly, M., Groop, L., and Lander, E. S. (2000) The common PPARgamma Pro12Ala polymorphism is associated with decreased risk of type 2 diabetes. *Nat. Genet*., **26,** 76–80.

[40] Hegele, R. A. (2000) Familial partial lipodystrophy: A monogenic form of the insulin resistance syndrome. *Mol. Genet. Metab*., **71,** 539–544.

[41] Cao, H., and Hegele, R. A. (2000) Nuclear lamin A/C R482Q mutation in canadian kindreds with Dunnigan-type familial partial lipodystrophy. *Hum. Mol. Genet*., **9,** 109–112.

[42] Shackleton, S., Lloyd, D. J., Jackson, S. N., Evans, R., Niermeijer, M. F., Singh, B. M., Schmidt, H., Brabant, G., Kumar, S., Durrington, P. N., Gregory, S., O'Rahilly, S., and Trembath, R. C. (2000) LMNA, encoding lamin A/C, is mutated in partial lipodystrophy. *Nat. Genet*., **24,** 153–156.

[43] Speckman, R. A., Garg, A., Du, F., Bennett, L., Veile, R., Arioglu, E., Taylor, S. I., Lovett, M., and Bowcock, A. M. (2000) Mutational and haplotype analyses of families with familial partial lipodystrophy (Dunnigan variety) reveal recurrent missense mutations in the globular C-terminal domain of lamin A/C. *Am. J. Hum. Genet*., **66,** 1192–1198.

[44] Agostini, M., Gurnell, M., Savage, D. B., Wood, E. M., Smith, A. G., Rajanayagam, O., Garnes, K. T., Levinson, S. H., Xu, H. E., Schwabe, J. W., Willson, T. M., O'Rahilly, S., and Chatterjee, V. K. (2003) Tyrosine agonists reverse the molecular defects associated with dominant negative mutations in human PPAR gamma. *Endocrinology*, **145,** 1527–1538.

[45] Savage, D. B., Tan, G. D., Acerini, C. L., Jebb, S. A., Agostini, M., Gurnell, M., Williams, R. L., Umpleby, A. M., Thomas, E. L., Bell, J. D., Dixon, A. K., Dunne, F., Boiani, R., Cinti, S., Vidal-Puig, A., Karpe, F., Chatterjee, V. K., and O'Rahilly, S. (2003) Human metabolic syndrome resulting from dominant-negative mutations in the nuclear receptor peroxisome proliferator-activated receptor-gamma. *Diabetes*, **52,** 910–917.

[46] Nolte, R. T., Wisely, G. B., Westin, S., Cobb, J. E., Lambert, M. H., Kurokawa, R., Rosenfeld, M. G., Willson, T. M., Glass, C. K., and Milburn, M. V. (1998) Ligand binding and co-activator assembly of the peroxisome proliferator-activated receptor-gamma. *Nature*, **395,** 137–143.

[47] Miles, P. D., Barak, Y., He, W., Evans, R. M., and Olefsky, J. M. (2000) Improved insulin-sensitivity in mice heterozygous for PPAR-gamma deficiency. *J. Clin. Invest.*, **105,** 287–292.

[48] Kubota, N., Terauchi, Y., Miki, H., Tamemoto, H., Yamauchi, T., Komeda, K., Satoh, S., Nakano, R., Ishii, C., Sugiyama, T., Eto, K., Tsubamoto, Y., Okuno, A., Murakami, K., Sekihara, H., Hasegawa, G., Naito, M., Toyoshima, Y., Tanaka, S., Shiota, K., Kitamura, T., Fujita, T., Ezaki, O., Aizawa, S., Kadowaki, T., et al. (1999) PPAR gamma mediates high-fat diet-induced adipocyte hypertrophy and insulin resistance. *Mol. Cell*, **4,** 597–609.

[49] Hu, E., Kim, J. B., Sarraf, P., and Spiegelman, B. M. (1996) Inhibition of adipogenesis through MAP kinase-mediated phosphorylation of PPARgamma. *Science*, **274,** 2100–2103.

[50] Camp, H. S., and Tafuri, S. R. (1997) Regulation of peroxisome proliferator-activated receptor gamma activity by mitogen-activated protein kinase. *J. Biol. Chem.*, **272,** 10811–10816.

[51] Rangwala, S. M., Rhoades, B., Shapiro, J. S., Rich, A. S., Kim, J. K., Shulman, G. I., Kaestner, K. H., and Lazar, M. A. (2003) Genetic modulation of PPARgamma phosphorylation regulates insulin sensitivity. *Dev. Cell*, **5,** 657–663.

[52] Shuldiner, A. R., Nguyen, W., Kao, W. H., Beamer, B. A., Andersen, R. E., Pratley, R., and Brancati, F. L. (2000) Pro115Gln peroxisome proliferator-activated receptor-gamma and obesity. *Diabetes Care*, **23,** 126–127.

[53] Schaffler, A., Barth, N., Schmitz, G., Zietz, B., Palitzsch, K. D., and Scholmerich, J. (2001) Frequency and significance of Pro12Ala and Pro115Gln polymorphism in gene for peroxisome proliferation-activated receptor-gamma regarding metabolic parameters in a Caucasian cohort. *Endocrine*, **14,** 369–373.

[54] Hamer, O. W., Forstner, D., Ottinger, I., Ristow, M., Bollheimer, L. C., Scholmerich, J., and Palitzsch, K. D. (2002) The Pro115Gln polymorphism within the PPAR gamma2 gene has no epidemiological impact on morbid obesity. *Exp. Clin. Endocrinol. Diabetes*, **110,** 230–234.

[55] Bluher, M., and Paschke, R. (2003) Analysis of the relationship between PPAR-gamma 2 gene variants and severe insulin resistance in obese patients with impaired glucose tolerance. *Exp. Clin. Endocrinol. Diabetes*, **111,** 85–90.

[56] Olansky, L., Marchetti, A., and Lau, H. (2003) Multicenter retrospective assessment of thiazolidinedione monotherapy and combination therapy in patients with type 2 diabetes: Comparative subgroup analyses of glycemic control and blood lipid levels. *Clin. Ther.*, **25(Suppl B),** B64–B80.

[57] Kletzien, R. F., Clarke, S. D., and Ulrich, R. G. (1992) Enhancement of adipocyte differentiation by an insulin-sensitizing agent. *Mol. Pharmacol.*, **41,** 393–398.

[58] Chawla, A., Schwarz, E. J., Dimaculangan, D. D., and Lazar, M. A. (1994) Peroxisome proliferator-activated receptor (PPAR) gamma: Adipose-predominant expression and induction early in adipocyte differentiation. *Endocrinology*, **135,** 798–800.

[59] Spiegelman, B. M. (1998) PPAR-gamma: Adipogenic regulator and thiazolidinedione receptor. *Diabetes*, **47,** 507–514.

[60] Cobb, J. E., Blanchard, S. G., Boswell, E. G., Brown, K. K., Charifson, P. S., Cooper, J. P., Collins, J. L., Dezube, M., Henke, B. R., Hull-Ryde, E. A., Lake, D. H., Lenhard, J. M., Oliver, W., Jr., Oplinger, J., Pentti, M., Parks, D. J., Plunket, K. D., and Tong, W. Q. (1998) *N*-(2-Benzoylphenyl)-L-tyrosine PPARgamma agonists. 3. Structure-activity relationship and optimization of the *N*-aryl substituent. *J. Med. Chem.*, **41,** 5055–5069.

[61] Burant, C. F., Sreenan, S., Hirano, K., Tai, T. A., Lohmiller, J., Lukens, J., Davidson, N. O., Ross, S., and Graves, R. A. (1997) Troglitazone action is independent of adipose tissue. *J. Clin. Invest.*, **100,** 2900–2908.

[62] Chao, L., Marcus-Samuels, B., Mason, M. M., Moitra, J., Vinson, C., Arioglu, E., Gavrilova, O., and Reitman, M. L. (2000) Adipose tissue is required for the antidiabetic, but not for the hypolipidemic, effect of thiazolidinediones. *J. Clin. Invest.*, **106,** 1221–1228.

[63] Hevener, A. L., He, W., Barak, Y., Le, J., Bandyopadhyay, G., Olson, P., Wilkes, J., Evans, R. M., and Olefsky, J. (2003) Muscle-specific Pparg deletion causes insulin resistance. *Nat. Med.*, **9,** 1491–1497.

[64] Norris, A. W., Chen, L., Fisher, S. J., Szanto, I., Ristow, M., Jozsi, A. C., Hirshman, M. F., Rosen, E. D., Goodyear, L. J., Gonzalez, F. J., Spiegelman, B. M., and Kahn, C. R. (2003) Muscle-specific PPARgamma-deficient mice develop increased adiposity and insulin resistance but respond to thiazolidinediones. *J. Clin. Invest.*, **112,** 608–618.

[65] Unger, R. H. (2002) Lipotoxic diseases. *Annu. Rev. Med.*, **53,** 319–336.

[66] Yamauchi, T., Kamon, J., Waki, H., Murakami, K., Motojima, K., Komeda, K., Ide, T., Kubota, N., Terauchi, Y., Tobe, K., Miki, H., Tsuchida, A., Akanuma, Y., Nagai, R., Kimura, S., and Kadowaki, T. (2001) The mechanisms by which both heterozygous peroxisome proliferator-activated receptor gamma (PPARgamma) deficiency and PPARgamma agonist improve insulin resistance. *J. Biol. Chem.*, **276,** 41245–41254.

[67] Okuno, A., Tamemoto, H., Tobe, K., Ueki, K., Mori, Y., Iwamoto, K., Umesono, K., Akanuma, Y., Fujiwara, T., Horikoshi, H., Yazaki, Y., and Kadowaki, T. (1998) Troglitazone increases the number of small adipocytes without the change of white adipose tissue mass in obese Zucker rats. *J. Clin. Invest.* **101,** 1354–1361.

[68] de Souza, C. J., Eckhardt, M., Gagen, K., Dong, M., Chen, W., Laurent, D., and Burkey, B. F. (2001) Effects of pioglitazone on adipose tissue remodeling within the setting of obesity and insulin resistance. *Diabetes*, **50,** 1863–1871.

[69] Akazawa, S., Sun, F., Ito, M., Kawasaki, E., and Eguchi, K. (2000) Efficacy of troglitazone on body fat distribution in type 2 diabetes. *Diabetes Care*, **23,** 1067–1071.

[70] Mori, Y., Murakawa, Y., Okada, K., Horikoshi, H., Yokoyama, J., Tajima, N., and Ikeda, Y. (1999) Effect of troglitazone on body fat distribution in type 2 diabetic patients. *Diabetes Care*, **22,** 908–912.

[71] Giusti, V., Verdumo, C., Suter, M., Gaillard, R. C., Burckhardt, P., and Pralong, F. (2003) Expression of peroxisome proliferator-activated receptor-gammal and peroxisome proliferator-activated receptor-gamma2 in visceral and subcutaneous adipose tissue of obese women. *Diabetes*, **52,** 1673–1676.

[72] Tsao, T. S., Lodish, H. F., and Fruebis, J. (2002) ACRP30, a new hormone controlling fat and glucose metabolism. *Eur. J. Pharmacol.*, **440,** 213–221.

[73] Yamauchi, T., Kamon, J., Waki, H., Terauchi, Y., Kubota, N., Hara, K., Mori, Y., Ide, T., Murakami, K., Tsuboyama-Kasaoka,

N., Ezaki, O., Akanuma, Y., Gavrilova, O., Vinson, C., Reitman, M. L., Kagechika, H., Shudo, K., Yoda, M., Nakano, Y., Tobe, K., Nagai, R., Kimura, S., Tomita, M., Froguel, P., and Kadowaki, T. (2001) The fat-derived hormone adiponectin reverses insulin resistance associated with both lipoatrophy and obesity. *Nat. Med.*, **7,** 941–946.

[74] Berg, A. H., Combs, T. P., Du, X., Brownlee, M., and Scherer, P. E. (2001) The adipocyte-secreted protein Acrp30 enhances hepatic insulin action. *Nat. Med.*, **7,** 947–953.

[75] Fruebis, J., Tsao, T. S., Javorschi, S., Ebbets-Reed, D., Erickson, M. R., Yen, F. T., Bihain, B. E., and Lodish, H. F. (2001) Proteolytic cleavage product of 30-kDa adipocyte complement-related protein increases fatty acid oxidation in muscle and causes weight loss in mice. *Proc. Natl. Acad. Sci. U.S.A.*, **98,** 2005–2010.

[76] Steppan, C. M., and Lazar, M. A. (2002) Resistin and obesity-associated insulin resistance. *Trends Endocrinol. Metab.*, **13,** 18–23.

[77] Steppan, C. M., Bailey, S. T., Bhat, S., Brown, E. J., Banerjee, R. R., Wright, C. M., Patel, H. R., Ahima, R. S., and Lazar, M. A. (2001) The hormone resistin links obesity to diabetes. *Nature*, **409,** 307–312.

[78] Way, J. M., Gorgun, C. Z., Tong, Q., Uysal, K. T., Brown, K. K., Harrington, W. W., Oliver, W. R., Jr., Willson, T. M., Kliewer, S. A., and Hotamisligil, G. S. (2001) Adipose tissue resistin expression is severely suppressed in obesity and stimulated by peroxisome proliferator-activated receptor gamma agonists. *J. Biol. Chem.*, **276,** 25651–25653.

[79] Savage, D. B., Sewter, C. P., Klenk, E. S., Segal, D. G., Vidal-Puig, A., Considine, R. V., and O'Rahilly, S. (2001) Resistin/Fizz3 expression in relation to obesity and peroxisome proliferator-activated receptor-gamma action in humans. *Diabetes*, **50,** 2199–2202.

[80] Hotamisligil, G. S., Shargill, N. S., and Spiegelman, B. M. (1993) Adipose expression of tumor necrosis factor-alpha: Direct role in obesity-linked insulin resistance. *Science*, **259,** 87–91.

[81] Moller, D. E. (2000) Potential role of TNF-alpha in the pathogenesis of insulin resistance and type 2 diabetes. *Trends Endocrinol. Metab.*, **11,** 212–217.

[82] Hotamisligil, G. S., Arner, P., Caro, J. F., Atkinson, R. L., and Spiegelman, B. M. (1995) Increased adipose tissue expression of tumor necrosis factor-alpha in human obesity and insulin resistance. *J. Clin. Invest.*, **95,** 2409–2415.

